# A Rare Case of Thoracic Pyogenic Facet Joint Infection

**DOI:** 10.1155/2019/8252986

**Published:** 2019-12-30

**Authors:** Tomoyuki Setoue, Jun-ichiro Nakamura

**Affiliations:** Saiwai Tsurumi Hospital, 1-21 Toyooka, Tsurumi, Yokohama, Japan 230-0062

## Abstract

Pyogenic facet joint infection is a rare but severe infection. The most common complaint on presentation is pain followed by fever, then neurologic impairment. While the lumbar spine is involved in the vast majority of cases presented in the literature, the case presented here occurred in the thoracic spine. The patient was a 48-year-old immune-competent female who presented with left back pain. Magnetic resonance imaging (MRI) indicated a facet effusion, paraspinal abscess, and epidural abscess in the level of 9th-11th thoracic vertebra. On the 6th day of treatment, she presented a neurological disorder and underwent decompressive laminectomy and surgical debridement. We observed immediate improvement as a result of the surgery.

## 1. Introduction

Pyogenic facet joint infection (PFJI) was first described in 1966 and was considered a relatively rare entity [1]. At present, about 40 cases of PFJI have been reported worldwide [2] but the exact incidence is unknown. Most PFJI cases occur in the lumber spine [3, 4]. We experienced a thoracic case, and because of the rarity of the location of infection, we considered it valuable to report.

## 2. Case Presentation

**Figure 1:**
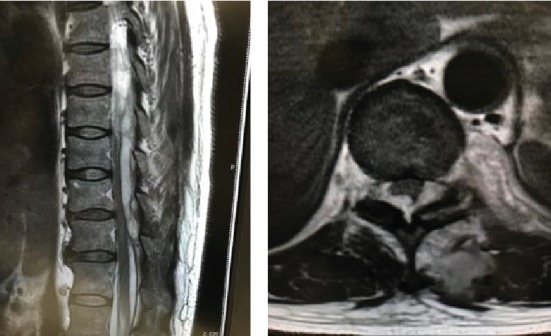
T2-weighted sagittal and axial view showed a facet effusion, paraspinal abscess, and epidural abscess in the level of 10th-11th thoracic vertebra.

A 48-year-old female patient presented with left back pain which began 3 days earlier. She described her pain as continuous, severe, precipitated by the smallest movement, and accompanied by fever in more recent days. There was no history of urinary incontinence, spinal trauma, or any other medical problem. She had no history of spine surgery, injections, or drug use. On clinical examination, we found her to be febrile and tachycardic, with the muscular strength in the lower limbs rating 5 according to the manual muscle test (MMT), normal sensations and reflexes, a negative Lasègue's test, and no pathological meningeal signs. In the first paraclinical test, we observed leukocytosis (1.920/mm^3^ of blood), neutrophilia (88%), an elevated level of C-reactive protein (23 mg/L), and normal urinalysis. The patient was hospitalized in internal medicine with a diagnosis of unknown fever. As the patient's condition deteriorated and our clinical suspicions increased, we elected to culture the patient's blood. In the culture, we discovered methicillin-sensitive *Staphylococcus aureus* (MSSA) and initiated the administration of intravenous antibiotics. On the 6th day of treatment, the patient complained of numbness in both limbs; her muscular strength was found to have slightly decreased on her right side. A MRI scan of the spine revealed a facet effusion, paraspinal abscess, and epidural abscess in the level of 10th-11th thoracic vertebra (Figure 1). Over the next few hours, the patient underwent decompressive laminectomy (at the level of Th 10/11) and surgical debridement. We posteriorly approached Th 9/10 and noted an abscess under the left multifidus muscle. We removed the necrotic muscle. We were able to recognize the melted articular capsule and cartilage of the left Th 10/11 facet joint and insert an elevator into the joint. Upon insertion, we were able to confirm that an abscess had spread to the surrounding tissue. We performed thorough debridement of all granulation tissue and irrigated the area with saline. Intraoperative cultures also revealed the presence of MSSA.

**Figure 2:**
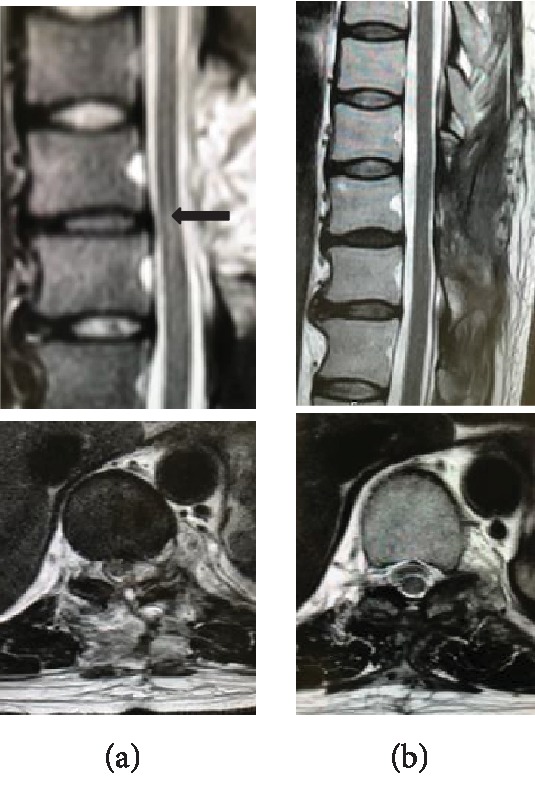
MRI taken on the 7th day after surgery (a) showed decompression of the spine and recorded a high signal in the spinal cord, indicating local ischemic changes of the spine at the Th 10 level ((a) arrow). The MRI taken 3 months after the surgery (b) showed no ischemic change or the facet effusion.

The patient was told to continue intravenous antibiotics for 6 weeks. We observed immediate improvement of the paresis and numbness as a result of the surgical decompression. A postoperative MRI taken on the 7th day after surgery showed decompression of the spine and recorded a high signal in the spinal cord, indicating local ischemic changes of the spine at the Th 10 level (Figure 2). The follow-up MRI taken 3 months after the surgery showed no ischemic change or the facet effusion (Figure 2); there was no recurrence of infection 1 year after surgery.

## 3. Discussion

Septic arthritis of the facet joint is a rare condition. Four retrospective reviews of case reports in the literature found that septic arthritis of the facet joint causes 4%–20% of pyogenic spinal infections, 86%–97% of which occur in the lumber spine [3–6]. It has been reported that there are some risk factors that predispose to infection in PFJI patients, but Narváez et al. have stated that more than 60% of PFJI patients show no systematic risk factors, indicating that spontaneous PFJI can also affect immune-competent patients, as was seen in our case [3].

While most cases are thought to occur via hematogenous spread [3, 4], there are some case reports in the literature where septic arthritis of the facet joint resulted from iatrogenic causes, including steroid injection and epidural catheterization [7–9]. These infections can also occur secondary to the spread of adjacent infections, such as spondylodiscitis, epidural or paraspinal abscess, psoas muscle abscess, and other intra-abdominal infections [5]. Based on MRI findings, we determined our case did not occur following spondylodiscitis. The blood culture indicated that the infection was hematogenous, but the origin was unknown. Although the pathogenesis of septic arthritis of the facet joint is not clear, previously reported epidemiology states that the most common causative organism of septic arthritis of the facet joints by hematogenous spread is *S. aureus*, as reported here [4, 6].

The pathophysiology of the epidural abscess is explained because of the narrow facet joint cavity, which facilitates the easy spread of infection to the epidural space (by rupture of the ventral aspect of the joint capsule) and to the paravertebral muscles (by rupture of the posterior aspect of the joint capsule) [1, 10]. Another mechanism of physiopathology of hematogenous spread to the extra facet has been reported in some studies [4, 5]. For example, Muffoletto et al. stated that hematogenous spread occurs via the branches of the posterior intercostal vessels [4].

In terms of the location of infection, PFJI of the thorax is very rare. If the epidural abscess occurs by the mechanism listed above, thoracic PFJI may easily accompany the abscess. This is because the thoracic facet is smaller than the lumber facet and the degeneration and thickening of the ligamentum flavum are less severe than that of the lumbar spine; therefore, the capsule may perforate earlier [11].

There are only two previous reports of thoracic PFJI, excluding iatrogenic cases [3, 12]. In one case, an immune-competent patient presented with an epidural abscess and bilateral extremity paresis and underwent immediate open facet joint drainage and decompressive laminectomy. On the other hand, primary thoracic epidural abscess has a relatively higher rate of occurrence and several cases describing it have been reported [13–15]. In terms of the order of infection occurrence, Muffoletto et al. suggested that the facet infections first occur independently and then lead to a secondary epidural or paraspinal muscle abscess [4]. Thus, some epidural abscesses that are believed to be of primary origin may in fact be complications of unrecognized PFJI [3].

Diagnosing this entity is difficult as it can behave as both a degenerative disease and spondylosis. The facet joint destruction can be seen on simple X-ray and CT images for about 1 month and 1 week after the onset, respectively. MRI is useful for early definitive diagnosis [4, 5]. It has been reported that signal changes in soft tissue appear 2 days after onset, but it often takes about 1 week to capture obvious changes [12]. The radiologic features seen in the MRI of the spine are joint effusion, periarticular bone marrow edema, and periarticular soft tissue edema with enhancement [4]. Therefore, it may be difficult to diagnose this condition when there is no associated paraspinal abscess. Conversely, the slight MRI findings of a facet infection may be missed if an epidural abscess appears extensively in the early stages of the disease, as was seen in the current case.

The treatment of choice for thoracic PFJI is conservative management with intravenous antibiotics, which is recommended for 4–6 weeks [5]. Intravenous antibiotics could be more effective in this case as compared to other joints because of the rich blood supply from a large number of collateral branches of arteries [5]. Surgical drainage with debridement is only reserved for patients with severe neurologic dysfunction. Severe neurologic complications are rare, occurring in less than 10% of the cases, and surgery is indicated in 26% of cases [3]. However, for the reasons mentioned above, thoracic PFJI may be more acutely and severely associated with paralysis than lumbar PFJI. Thus, surgical delay may cause severe paralysis in thoracic PFJI cases, which must be kept in mind by clinicians.
